# Studying stimuli and smoking behaviors among self-identified gifted smokers and strategies for customizing cessation support

**DOI:** 10.18332/tid/143323

**Published:** 2022-03-08

**Authors:** Danae Deligeorges, Michele Kane, Paraskevi A. Katsaounou

**Affiliations:** 1 School of Medicine, National and Kapodistrian University of Athens, Athens, Greece; 2 The Center for the Advancement of Noesis, Athens, Greece; 3 Special Education Department, Northeastern Illinois University, Chicago, United States; 4 Department of Pulmonary Medicine, National and Kapodistrian University of Athens, Athens, Greece; 5 Pulmonary and Respiratory Department of Intensive Care Medicine, Evangelismos Hospital, Athens, Greece

**Keywords:** smoking cessation, well-being, gifted, smoking triggers, smoking stimuli

## Abstract

**INTRODUCTION:**

The aim of the study was to examine smoking habits and triggers among self-identified gifted (inner experiences and awareness qualitatively different from the norm in terms of asynchronous development, advanced cognitive abilities, and heightened intensity) adults in order to customize smoking cessation support.

**METHODS:**

A total of 123 participants were enrolled through Facility-Based and Snowball Sampling. Quantitative analysis focused on the relationship between overexcitabilities, nicotine dependence, motivation to quit, and smoking triggers, using the following questionnaires: the Overexcitability Questionnaire (OEQ-II) an indicator of areas of overexcitability, a heightened response and lowered threshold to stimuli; the Fagerström Test for Nicotine Dependence; a Cessation Motivation Questionnaire; and a Smoking Triggers Questionnaire. Qualitative analysis drew on six semi-structured interviews. Participant experiences of the relationship between smoking behaviors and experiences of multiple areas of overexcitability were analyzed using process coding.

**RESULTS:**

The quantitative data indicate that self-identified gifted smokers who rate themselves as having 3–5 ‘High’ or ‘High Average’ overexcitabilities (OEs), are less likely to smoke in response to pattern, social, and addiction focused triggers than those self-reporting as having 0–2 OEs (Fisher’s exact test, p<0.1). In the interviews, we observed a high level of complexity in stimulation and smoking behavior, with all interviewees explicitly connecting their smoking habits with experiences of overexcitability. Two interviewees had given up smoking prior to the research and of the four who still smoked, three quit within a few days of the interview.

**CONCLUSIONS:**

We found that psychometric testing and interviews designed to increase self-knowledge about the relationship between overexcitabilities and triggers for smoking have the potential to improve outcomes for smoking cessation among the gifted.

## INTRODUCTION

Nicotine use, abuse, and addiction among the gifted is an understudied area despite evidence of their heightened responses and lowered thresholds to environmental stimuli, which have been shown to be connected to smoking habits as early as 1938^1^. The only recent study that examined smoking behaviors in relation to high IQ (2003) using data from the Scottish Mental Survey and the Midspan studies was conducted in the 1970s. The results showed that adults tested at 1 standard deviation (SD) above the mean for IQ, were 19% more likely to quit smoking, but noted the need for research on the factors that influence cessation^2^. There was no significant difference in the likelihood of starting smoking between smokers with average IQ and 1 SD above the average^2^. As far as we know, there is no other study that has been undertaken to examine the causes of smoking in the gifted population or the relationship between intrinsic traits, inner experience, overexcitabilities, and smoking – rather than simply correlating IQ with quantitative data on usage and cessation. Giftedness in not merely an IQ score^3^.

While this and previous studies on giftedness, substance use, and addiction, used childhood IQ as an indicator^4-6^, psychometric tests have been developed over the past 30 years to measure intrinsic traits. Therefore, the implications of inner experience for areas of behavior such as smoking and addiction can be directly studied. In our exploratory study, we examine the experience of overexcitabilities in the gifted population to better understand the implications for smoking behaviors and cessation. Based on recent research within the gifted population into inner experience^1,7-9^, we hypothesize that there is a qualitative difference in smoking and cessation behaviors and needs amongst smokers with multiple overexcitabilities, defined here as 3–5 overexcitabilities, as opposed to smokers with 0–2 overexcitabilities.

Looking at the teenage period when many smokers also start, a study of alcohol use among gifted young people suggested that they are as vulnerable to conforming to peer norms around substance use as the non-gifted. However, they were susceptible for different reasons: to compensate for the social price of their academic abilities or to mask their giftedness to avoid possible rejection due to stigmatization^4^. The study used aptitude tests, achievement, and teacher checklists as identifiers, and while they used primarily quantitative analysis, the behavioral, social, and psychological profiles of the participants were examined^4^.

The effects of nicotine on the body and the nature of its use are very different from those of alcohol; however, the context of the adolescent experience is relevant, as this is the period when first use commonly occurs^10^. There is evidence that many gifted individuals experience loneliness^4,11^; that they often feel misunderstood or difficulties relating with others^11^; and that their perceptual, emotional, sensual, and psychomotor experiences are particularly intense^1,11,12^. The relationship between these experiences, smoking habits, and cessation, has not been examined and is a significant factor in ensuring appropriate smoking cessation interventions.

Loneliness has also been identified as a factor correlated with higher nicotine consumption among smokers more broadly^13,14^. Gücük et al.^14^ studied smoking cessation support using interviews and psychometric tests to examine sociocultural and wellbeing factors in smoking behavior across 765 participants. They found that the mean loneliness scores were higher for smokers than non-smokers and noted the effect of loneliness on wellbeing, health, and self-esteem. In addition to emphasizing the role of family and the social environment, they suggested it may be helpful to customize support for smoking cessation based on psychometric evaluations^14^. Given that many gifted individuals experience heightened intensity and loneliness due to having difficulties interacting with others, making them susceptible to smoking, cessation research tailored to this population is particularly important. Currently, psychometric evaluations and approaches to motivational cessation interviews do not address their different needs. Our research into self-identified gifted smokers and overexcitabilities provides an initial trial of a well-established psychometric test from the gifted field^15^ and person-centered psychopedagogical interviews using an open, non-motivational approach to build self-knowledge as a part of smoking cessation support. This approach is based on psychiatrist Dabrowski’ s personality theory, the theory of positive disintegration, and established approaches to counselling the gifted, with a focus on facilitating selfknowledge and respecting the individual’s capacity for discernment and self-analysis^16^. Through analysis of these interviews, we examine causes and triggers of smoking, and barriers to cessation in this population.

The definition of giftedness is contentious; yet, worthy of exploration. While academic success, high IQ or eminence are often used as indicators of giftedness, these have been shown to have significant limitations due to their dependence on socioeconomic and cultural factors^17,18^ and the lack of attention to intrinsic potentiality and complexity^3^. It is essential in smoking cessation support to recognize the unique and complex needs of those who have advanced cognitive abilities and overexcitabilities, as their inner experiences and awareness are qualitatively different from the norm^19^. Giftedness is therefore defined here using the Columbus Group approach: in terms of asynchronous development, advanced cognitive abilities, and heightened intensity^3^.

Our approach draws on research suggesting that not everyone is vulnerable to developing substancerelated disorders, although substance use may function as a precursor to addiction for some^20^. We proceed with the assumption that smoking behavior has physiological, behavioral, psychological, and cognitive attributes that vary between individuals^21^.

The distinction between motivation to seek or use substances associated with ‘conditioned stimuli’, and motivation associated with reinforcement through dopamine transmission in the nucleus accumbens^14^ is particularly relevant to the gifted population, whose response to stimuli is already heightened. Evidence that habitual behavior is elicited automatically by conditioned stimuli suggests that smoking cessation support needs to address the impact of environmental stimuli that have become conditioned as triggers. Tackling physiological and psychological dependence by preventing initiation of the habit as well as supporting reduction and cessation is also essential. For this reason, we emphasized triggers for smoking as part of both psychometric testing and the interview process. We adapted the list of common triggers for smoking, provided by the United States National Cancer Institute^22^ and Dabrowski’s analysis of complex triggers^1^ relating to overexcitabilities, into a questionnaire to identify triggers. This underpinned a quantitative analysis of the relationship between overexcitabilities and triggers, using the following categories: triggers related to addiction, social smoking, patterns in behavior, emotional triggers, and complex triggers. The questionnaire also facilitated the interview process, building participants’ understanding of their patterns of behavior and the complex triggers unique to them.

As we talked to participants in initial group meetings, it became clear that they wanted a more socio-emotional approach to quitting: to be understood and to be given a reason beyond health. When asked how willing they were to participate in smoking cessation using pharmaceutical support, the majority declined, although those with underlying medical conditions said they would consider it. We faced a significant challenge in customizing provision for the gifted, in that many viewed traditional approaches as unhelpful (pharmaceutical support, motivational talks or interviews^23^, and information about health risks and benefits). We therefore trialed research tools that identify specific traits related to heightened sensitivity and intensity of response to stimuli, drawing on Dabrowski’s theory of positive disintegration – which was named due to its positive contribution to inner growth^24^.

This conceptual framework is a non-ontogenetic theory of personality development, based on research into the psychology and traits of individuals with high developmental potential. It provides a reconsideration of how periods of psychological maladjustment and inner conflict contribute to personality development. Dabrowski believed that everyone is shaped by their developmental potential, which is influenced by three factors: 1) specific abilities and general intelligence; 2) overexcitabilities (psychomotor, sensual, imaginational, intellectual, and emotional); and 3) the capacity for inner growth and self-determination^25^. As one of these factors, the strength and number of the overexcitabilities contribute to both the potential for development^26^ and to a heightened intensity of inner experience. Of particular significance to this study, however, is Dabrowski’s research into how the overexcitabilities function as a means of processing the excessive emotional tension they create^24,27^. Tension or heightened responses to stimuli in one area can be processed or released through another^1,24^. Dabrowski relates this to the act of smoking as a substitute action for other unacceptable forms of release of emotional tension^1,23,24,28,29^. The focus on personality development in Dabrowski’s work is also of significance as the shift into self-knowledge and the associated periods of maladjustment are additional contextual factors for the gifted, who often experience high levels of overexcitability across multiple areas^30^. The emergence of self-understanding, independent self-analysis, reflection, and introspection were particularly relevant to this study in designing our approach to the interviews.

Drawing on Dabrowski’s theory, we therefore examine: 1) how the unique experiences of multiple, combined overexcitabilities create barriers to smoking cessation – the heightened intensity of experience in everyday life can create an urgent need to soothe oneself using any means available instead of processing the experience; 2) How the unique experiences of multiple, combined overexcitabilities contribute to self-knowledge and the understanding of the strong, reactive responses to daily stimuli; and 3) strategies for encouraging cessation by providing support services that recognize and understand smokers who may be experiencing overexcitabilities, avoiding possible mental health misdiagnoses.

## METHODS

### Study design and setting

Within this mixed-methods study, we used both quantitative and qualitative data contributing to the understanding of how overexcitabilities are connected to smoking triggers and behaviors, and these approaches were trialed as tools for smoking cessation support. We approached participants through a talk on nicotine dependence called ‘Health Wins’, a collaboration between the Center for the Advancement of Noesis and the Department of Smoking Cessation of Evangelismos Hospital in Athens, and through gifted programs run through the Center for the Advancement of Noesis in Greece and Denmark.

We worked with volunteer participants. Most of them were Greek or Danish, and a few were British or American, and thus we decided to create two groups: Greek and non-Greek, in order to observe any potential differences, for example, due to failures in implementation of anti-smoking laws in Greece in contrast with other countries. The participants were limited to smokers aged 18–70 years that self-identified as gifted; this population was reached through the Center for the Advancement of Noesis and Gifted Children Denmark. Our approach to recruiting participants takes account of the ‘hard to reach’ nature of this population; the resulting limited means of identifying them; and the stigma associated with identifying as gifted^31-33^. Drawing on Shaghaghi, Bhopal & Sheikh’s review of sampling approaches for hard-to-reach populations, we combined Facility-Based Sampling (FBS) and Snowball Sampling to reach suitable participants, rather than a systematic or probability based approach. They signed consent forms, and the study was approved by the Evangelismos Hospital ethics committee (protocol number 117). Data were obtained from the 123 participants through questionnaires and semi-structured interviews; 72 completed at least three questionnaires; 62 completed all the questionnaires; and 6 participants with three or more overexcitabilities were invited to participate in interviews. Supplementary file Table 1 provides an overview of the sample.

**Table 1:** Levels of dependence according to FTND scores among self-identified gifted smokers, Greece (N=132)

*Characteristics*	*Categories of dependence*	*%*
Greek all levels of OE	Mild (0–3)	40.4
	Moderate (4–6)	59.6
	High (>7)	0
Greek 0–2 OEs	Mild (0–3)	35.7
	Moderate (4–6)	64.3
	High (>7)	0
Greek 3–5 OEs	Mild (0–3)	41.9
	Moderate (4–6)	58.1
	High (>7)	0
Non-Greek all levels of OE	Mild (0–3)	13.3
	Moderate (4–6)	87.7
	High (>7)	0
Non-Greek 0–2 OEs	Mild (0–3)	33.3
	Moderate (4–6)	66.7
	High (>7)	0
Non-Greek 3–5 OEs	Mild (0–3)	0
	Moderate (4–6)	100
	High (>7)	0
Whole sample, all levels of OE	Mild (0–3)	34.7
	Moderate (4–6)	65.3
	High (>7)	0
Whole sample 0–2 OEs	Mild (0–3)	35
	Moderate (4–6)	65
	High (>7)	0
Whole sample 3–5 OEs	Mild (0–3)	34.6
	Moderate (4–6)	65.4
	High (>7)	0

OE: overexcitability

### Questionnaires

We used four questionnaires across the sample: the Fagerström Test for Nicotine Dependence (FTND)^34,35^, Cessation Motivation Questionnaire^36^, Smoking Triggers Questionnaire, and the Overexcitability Questionnaire (OEQ-II)^15^. The questionnaires were followed by six semi-structured interviews and post interview feedback.

*Fagerström Test for Nicotine Dependence (FTND)*

The FTND is a standard instrument for assessing levels of nicotine addiction. It uses six questions to score dependence from 0 to 10 points. We used three categories of dependence: low (0–3), moderate (4–6), and high (7–10).

*Cessation motivation questionnaire*

The Cessation Motivation Questionnaire uses 16 questions with a three-point scale, providing an indication of levels of motivation:

1. Mostly has decided to stop smoking;
2. Mostly is considering stopping smoking; and
3. Mostly is not considering stopping smoking.

In addition to providing data, the FTND and Cessation Motivation Questionnaire were used to ensure that interviews involved participants across ranges of levels of dependence and motivation to quit, and to facilitate discussion within the interviews.

*Overexcitability Questionnaire (OEQ-II)*

The OEQ-II provides a measure of five areas of heightened responses and lowered thresholds to stimuli, referred to as overexcitabilities. The questionnaire is easily administered and scored using 50 questions spread across the five overexcitabilities^15^. It uses a five-point Likert scale from 1=‘not at all like me’ to 5=‘very much like me’. There is one score for each overexcitability category: Low, Low Average, Average, High Average, and High. Individual cutoffs for each overexcitability were established through previous studies of a standardized population (N=887)^37^. We used High Average and high as indicators that an individual had a particular overexcitability. We used the OEQ-II to build self-knowledge in the interviews, and as the basis for quantitative analysis of the relationship between multiple overexcitabilities and smoking habits. The OEQ-II would be of benefit in treating smokers who experienced heightened stimulation, whether or not they identify as gifted.

*Smoking triggers questionnaire*

We adapted into a short indicative questionnaire, common triggers for smoking according to the United States National Cancer Institute^22^ and Dabrowski’s analysis of complex triggers related to overexcitabilities^24^. The questionnaire provides 40 statements describing triggers for smoking across five categories: emotional, social, pattern, addiction, and complex. Participants were asked to tick all that applied.

### Analytical methods

In the quantitative analysis, we assessed levels of dependence, motivation, and triggers for smoking across the sample, and we examined the correlation between these and the presence or absence of multiple overexcitabilities. Significance was set at a level of p<0.1 using the Fischer’s exact test to indicate whether there is a justifiable need for further quantitative research in this area. However, the sample was too small to draw broader conclusions across the population from these data.

In the qualitative analysis, we used an inductive approach, involving process coding to identify patterns of behavior^38^ identified within the participants’ interpretations of their smoking habits and relationships with overexcitabilities. We coded these in relation to overexcitabilities, triggers, motivation to quit, and dependence. Our focus was on analyzing their accounts of experiences relating to overexcitabilities, smoking habits, reasons for smoking, motivations, and experience of smoking, including how they started smoking, giving a sense of change over time in their experience and management of intensities. Additionally, we identified areas of behavioral complexity, which in some cases also underpin emotional or pattern-based smoking behaviors; we identified the relationship between smoking triggers, habits and experience of overexcitabilities; and how participants managed their overexcitabilities before they started smoking. This provided a way to identify patterns and common themes across the interviews and to clarify where the qualitative and quantitative results converge and diverge.

The interviews focused on participants with three or more overexcitabilities to understand how the interrelation of multiple areas of overexcitability affects the complexity of patterns of behavior in smoking. Analysis also drew on post-interview feedback to understand the impact of the interviews on the participants.

## RESULTS

### Quantitative results

The examination of dependence and motivation to quit was based on the 72 participants who completed the first three tests. The analysis of the triggers was based on the 62 participants who completed all four of the tests. Our primary aim in the quantitative analysis was to understand if there were significant differences in smoking behaviors and triggers between participants with multiple overexcitabilities and those with 0–2.

### Levels of dependence

There were no differences in dependence between participants with 0–2 and 3–5 overexcitabilities. The level of dependence in this sample was unusually low, with no participants reporting high dependence (Table 1). The mean FTND score (±SD) for the entire sample was 4.15 (±1.33), no statistical difference (p=0.0687) was noted between the Greek sample (4.12±1.38) and the non-Greek sample (4.27±1.16).

### Motivation to quit

A large proportion of participants self-reported as ‘considering stopping smoking’. This was the case across the entire sample, with no significant differences between categories. A higher proportion of participants with 3–5 overexcitabilities were sure that they wanted to stop smoking than those with 0–2 overexcitabilities; however, this difference was not statistically significant (Table 2).

**Table 2:** Comparison of motivation to quit by levels of overexcitability (N=123)

*Characteristics*	*Categories*	*%*
Greek all levels of OE	A	19.3
	B	50.9
	C	19.3
	A/B	3.5
	B/C	7.0
Greek 0–2 OEs	A	0
	B	64.3
	C	21.4
	A/B	0
	B/C	14.3
Greek 3–5 OEs	A	25.6
	B	46.5
	C	18.6
	A/B	4.7
	B/C	4.79
Non-Greek all levels of OE	A	13.3
	B	46.7
	C	26.7
	A/B	0
	B/C	13.3
Non-Greek 0–2 OEs	A	16.7
	B	50
	C	16.7
	A/B	0
	B/C	16.7
Non-Greek 3–5 OEs	A	11.1
	B	42.2
	C	33.3
	A/B	0
	B/C	11.1
Whole sample all levels of OE	A	18.05
	B	50
	C	20.83
	A/B	2.77
	B/C	8.33
Whole sample 0–2 OEs	A	5
	B	60
	C	20
	A/B	0
	B/C	15
Whole sample 3–5 OEs	A	23.07
	B	46.15
	C	21.15
	A/B	3.846
	B/C	5.76

A: decided to stop smoking. B: considering stopping smoking. C: not considering stopping smoking. A/B: decided/considering. B/C: considering/not considering.

### Smoking triggers

In identifying triggers, we found that participants with 3–5 overexcitabilities had significantly lower response rates to statements about addiction, social situations, and patterns of behavior, than those with 0–2 overexcitabilities, but had higher response rates to statements about emotional and complex triggers. The differences in complex and emotional triggers were not statistically significant, but they are noted observationally for future study. We calculated the number of triggers ticked in each category across the group as a proportion of the number of statements provided (Table 3). For participants with 3–5 overexcitabilities, triggers regarding addiction were the least important, with social and emotional triggers being the most important. For participants with 0–2 overexcitabilities, triggers in order of importance were social, pattern, addiction, emotional, and complex.

**Table 3:** Differences between smokers with 0–2 OEs and 3–5 OEs in frequency of trigger categories identified

*Triggers*	*3–5 OEs % of possible positive responses*	*0–2 OEs % of possible positive responses*	p*
Social	41.0	52.4	0.0797
Emotional	38.2	33.3	NS
Pattern	29.4	38.1	0.0805
Complex	26.6	19.8	NS
Addiction	24.4	33.7	0.0701

* Significance using Fisher’s exact test; significant at p<0.1. NS: not significant. OE: overexcitability.

### Qualitative analysis

In the first part of the interviews of the six participants with three or more overexcitabilities who were invited to participate, we focused on facilitating increased self-knowledge and understanding of participants’ experiences of overexcitabilities. An overview of key comments selected from across the interviews is provided in Table 4. These were taken from participants’ responses to the question: ‘How do you experience [named overexcitability]?’, after a brief description from the interviewer based on the results of their OEQ-II questionnaire.

**Table 4:** Interview descriptions of experiences of overexcitabilities from the qualitative interviews (N=6)

	
Psychomotor OE	When something intense happens I need to move. Now in the interview I’m moving my hands to stabilize my energies to participate and concentrate. I want to get involved in things, I’m constantly moving to find calm. When I sit I need to move to calm myself. Even when I’m chilling I’m doing projects on my phone. I’m a crazy doer. When I’m sitting I jiggle my foot. People think it’s a tic. To have a coffee sitting down is torture.
Sensual OE	The senses aren’t tools to live, they are something to experience. I get enjoyment from the combination of senses. Because the intensity created by an image is so great it affects my sense of taste. When I see something beautiful in nature I want to drink wine to enjoy the moment more. One of favorite smells is the mouth of cats or any dirty object. I was also chewing clothes if they smelled of washing liquid. Food is sexy, I don’t eat jut to be full, but to enjoy it. The smell of someone is important for me to enjoy their company.
Intellectual OE	It’s good and bad being over-analytical, I can't be calm, or let go, I don't have limits. I lose my sense of moderation. It isn't something I control, my mind is not satisfied. It's pleasant to learn new things, but without limits, it becomes a burden and it gets me stuck. I put order into the chaos, but when I arrive at something specific, I pull it apart into chaos again. I have so many specialities that I don't know anymore what I am. My favorite question is why. Until there's nowhere to go or I have an answer. This annoys people around me, but I want to understand, to do things right and to understand all the details. I lose energy because my brain won't stop thinking. Yoga and meditation helps. My motor works quickly and demands answers.
Imaginational OE	I hope I don't seem paranoid! I simply escape into a visual reality. When its negative it isn't pleasant but when it's positive it is enjoyable. I can't interrupt or invite it. They can last three seconds but feel like whole days. After I process and filter it and continue. I don't know how it's triggered, let's say I think something like a dragon then a story starts where there are dragons. This can last hours. But in everyday life, in the back of my mind when I am angry or argue, I escape thinking my dragon will save me. I was imagining violence and my body was bruised. Dreamy! Every evening I make infinite scenarios, to feel good and to live. I am positive and I want to do everything even if it doesn't happen in the end. I go straight into what I Imagine. It might happen but if it doesn't I still lived it all.
Emotional OE	I have huge empathy for someone else, especially for difficult emotions, it’s like I have a responsibility to solve it, like I live it myself. I am as affected as much as them, to the same limit. Because my emotions are very intense, they explode in movement to feel that I can stand to live them. Yes, I can't control emotions. I cry from an advert, really the advertisers do very good work. I can't watch the news for anything, because I live things very intensely. Other people's problems are my problems. Other people's joys are my joys. For small things, I rejoice like a child. I stop eating for days from sadness. This characterizes me more than anything. In all emotions, I live them intensely and cry often. Whether I see something lovely or something awful, still I cry.

Table 4 provides an insight into the range of experiences of each overexcitability and areas of commonality in experience. Most participants had positive experiences of sensory overexcitability – enjoying the pleasure of sensory stimuli rather than experiencing them as overwhelming. It was particularly notable that there was a strong focus across the interviewees on taste and smell. The other overexcitabilities were experienced with a greater mix of positive and negative effects, but emotional overexcitability was most commonly cited where difficult or negative experiences existed. All participants in the qualitative research had a high level of emotional overexcitability; the intensity of emotions and lower threshold for emotional response to stimuli came across very clearly in the interviews: ‘I cry from an advert’, for example.

The second half of the interview focused on smoking habits, experiences, and triggers. We analyzed the transcripts using process coding, focusing on behaviors and experiences identified within the participants’ interpretations of their smoking habits and relationships with overexcitabilities. We found that all interviewees started smoking regularly in their late teens. All explicitly related their smoking behaviors to the overexcitabilities they experienced and gave similar explanations of how it feels while smoking, focusing on the reduction of stress and emotional intensity, concentration, and feeling lighter. Five in six participants stated that one of the reasons for smoking was to deal with their emotions (Table 5).

**Table 5:** Interview responses coded from transcripts of the qualitative analysis (N=6)

*Participant*	*OEs*	*Do you find OES relevant to smoking habits?*	*Cigarettes per day*	*Age when you became a systematic smoker*	*How did you start smoking?*	*Why did/do you smoke?*	*How did you handle OEs before smoking?*	*How do you describe life as a smoker?*	*How do you feel before you light a cigarette?*	*How does it feel while smoking?*	*Reasons to quit*
No 9 mild smoker decided to quit	Sensual Psy/motor Emotional	YES	0–5	16–18	With friends	To fit in To deal with emotions To soothe the pain Stress	Crying a lot Swallowing too much pain Closed	I am more talkative and I share my problems with others	Stressed Emotional	I smoke my emotions away Concentrate Lighter	Motherhood
No 16 mild smoker considering quitting	Sensual Psy/motor Emotional Intellectual Imagin/al	YES	0–5	17	With friends	To stop moving For the taste To calm down	Restless Constantly moving Working out hard Tense	I can enjoy the company of my friends and I can sit and chill	Stressed Emotional	I smoke my emotions away Concentrate Lighter	Aesthetics Not good for my health
No 4 mild smoker doesn't want to quit	Sensual Emotional Intellectual	YES	0–10	19–20	With friends	To be social To deal with emotions Stress	Distant Antisocial Over-thinking	I can be a good friend and handle being out with a lot of people	Stressed Emotional	smoke my emotions away Concentrate Lighter	No reason
No 86 moderate smoker decided to quit	Sensual Psy/motor Emotional Intellectual	YES	20–40	18	With friends	To fit in To soothe the pain To deal with emotions	Depressed Aggressive Distant Restless Working out hard	Less painful Distracted from difficulties Can cope with reality	Stressed Emotional	I smoke my emotions away Concentrate Lighter	Health Aesthetics
No 22 moderate smoker considering quitting	Psy/motor Emotional Intellectual	YES	20–30	19	With friends	To calm down To be social To deal with emotions Stress	Over-thinking Stressed	Less outbursts Not gaining weight More social Calmer	Stressed Emotional	I smoke my emotions away Concentrate Lighter	If someone convinced me why I should stop smoking
No 37 moderate smoker doesn't want to quit	Sensual Emotional Intellectual Imagin/nal	YES	5–15	16–18	With friends	For a break To deal with emotions To concentrate Stress	I couldn't handle myself and life	I can cope with life Concentrate Calmer Not gaining weight	Stressed Emotional	I smoke my emotions away Concentrate Lighter	No reason

While it is not possible to include the full case study analysis here, we provide an overview of findings with key illustrative examples and quotations below. Each participant had unique experiences of handling their overexcitabilities before smoking and of how smoking helped them cope. There was a high level of complexity in behavior and understanding across all the responses, and among those in the category ‘mildly dependent’, there was also greater intentionality in smoking use. It appears that they had remained at the level of use without progressing to addiction^20^, but they believed that they could not quit because of the way that smoking was connected to complex emotional and behavioral patterns.

Participant 16 (mild smoker, 0–5 cigarettes/ day, considering quitting) scored high on addiction triggers in the triggers questionnaire despite having a low dependence on the FTND. She believed she was addicted because she could not quit. The process of analyzing her patterns and behaviors shifted this perception and she quit smoking within a week of the interview. Her quotation illustrates the complexity of smoking habits, the relationship with sensual and psychomotor overexcitabilities, and the expression of emotion through them:

*‘Smoking is connected with stabilizing what I'm experiencing — I can drink something, speak, understand this is the hour that I relax. After 3, I can smoke, not before. I can't smoke all the hours of the day. I don't need to. I don't have the appetite. I don't like it. It has to be in combination with something – I need to smoke with something specific – something sweet, like sweet coffee or alcohol or juice. I can't smoke drinking tea or water. If something very intense happens, I can smoke earlier, in the morning, but not usually. In that situation – it helps with the psychomotor, to calm down. I can stop to think and not have physical actions straight from a feeling.’*

Of the three mildly dependent smokers interviewed, Participant 9 informed us she had already quit smoking due to becoming a mother, and according to post interview feedback, both Participant 16 and Participant 4 quit smoking since the interview. Participant 4 quit despite having no prior intention to do so. They cited understanding their overexcitabilities and self-knowledge due to the interview as enabling them to stop.

We found that those with a moderate level of dependence, however, focused more on the need for a cigarette, and appeared to have shifted from intentional to compulsive use:

Participant 22 (20–30 cigarettes/day, considering quitting) said:

*‘When I'm angry with the kids, I say give me five minutes to calm down and I light a cigarette, and after I am calmer. And in the morning also … I don't want to speak to anyone until I've had a coffee and a cigarette. I am too easily irritated.’*

Participant 86 (20–40 cigarettes/day, has decided to quit) said:

*‘I agreed with myself that until 30, I could smoke as much as I want. And that's what I did. I couldn't stop for anything. The pills didn't help me to stop, they made me feel worse ... It helped a lot that decision I had taken.’*

Participant 37 (5–15 cigarettes/day, is not considering quitting):

*‘I have tried – a couple of months when I didn’t smoke. I put weight on – that and the intensity was difficult when I tried to stop.’*

Of the moderate smokers, Participant 86 had already quit and found it extremely difficult, stating:

*‘Nothing covered the emotional emptiness for two or three years and I was crazy. After a while it was selfknowledge which threw light and calm in the darkness I felt.’*

Participant 37, despite originally having a low motivation to quit in the motivation questionnaire, stopped smoking the day after the interview, also citing self-knowledge and understanding of the triggers and OEs. Participant 22 has had no change to her smoking habits since the interview. She stated she is going through a period of emotional distress and would not find stopping smoking psychologically beneficial despite the wider health benefits.

What is also notable across all the interviews is the connection with specific stimuli. This is clear in the quotation from Participant 16 and recurs across interviews. Conditioned stimuli^20^ are usually neutral, such as the sight of a lighter. However, when the stimuli for smoking are complex, such as fully integrated emotional, sensual and psychomotor intensities in combination with patterns (seeing a lighter, having a break at work), the challenge of reducing exposure to stimuli is significant.

## DISCUSSION

We found that the reasons for smoking and the challenges for smoking cessation were qualitatively different among self-identified gifted individuals with multiple overexcitabilities. Adjustments to the provision of smoking cessation support may be required for this population in both the Greek and non-Greek groups. Cultural differences do not seem to be significant based on this sample, however a larger comparative study is needed.

Our key findings are as follows. There were differences in the triggers for smoking between smokers with multiple overexcitabilities and smokers without them at a level of significance that indicates the need for further research. Moreover, the combination of intellectual, emotional, imaginational, sensual, and psychomotor overexcitabilities contributes to complex patterns of smoking that are unique to each individual. Finally, three of the four smokers in the interviews who had not previously quit reported quitting smoking soon after completing our cessation process.

We suggest that to unpack the complexity of triggers and patterns of smoking identified in gifted smokers, interviews that facilitate self-understanding are likely to be effective. There was a visible ‘Aha!’ moment in each interview, where the participant saw and understood their behavior and discovered the connections between their overexcitabilities and smoking habits. The OEQ-II is therefore useful both in identifying overexcitabilities and facilitating selfknowledge about complex combinations of stimuli. This can facilitate independent decision-making about strategies for cessation.

It is important to note that the interviews were conducted without a motivational element. The benefits of smoking that interviewees experienced were recognized and not denied, and participants were already well-informed of the health risks, having attended our talk at the beginning of the research process. This approach to building self-understanding was used because those who experience multiple overexcitabilities are more likely to respond to support that respects their own values and decision-making^39^. We found this approach very well-received in the trial.

Three of the four interviewees who were still smokers during the interviews, reported in their feedback approximately a month apart since the interviews were completed, that they had not resumed smoking after the end of the interview, stating that once they understood why they smoked, they were able to make decisions to use replacement activities in addressing elements of the pattern or to reduce feelings of intensity. No interviewee used pharmaceutical treatment. However, the considerably lower level of dependence observed in this sample than in comparable studies in Greece^40,41^ may be a factor in cessation success. It is not clear whether this lower dependence can be attributed to differences in traits of the gifted or differences in the sampling approach. Comparable studies have enrolled participants through attendance at smoking cessation clinics, where the level of dependence may be higher than average.

Early intervention is also critical. Like the majority of smokers^10^, the gifted participants began smoking in their teens. The period of adolescence when young people want to ‘fit-in’ with their peers is significant for the gifted due to the feelings of difference and loneliness they often experience^4-11^. Recognition of the complexity of decisions about smoking as well as better support for young people experiencing heightened intensities may also be useful approaches for gifted adolescents.

For smoking cessation provision more broadly, identifying smokers who experience heightened responses to stimuli and adjusting support in response, are likely to improve cessation rates. A substantial number of people could benefit from using this approach.

### Strengths and limitations

This is the first study creating and completing an initial trial of a methodological model which motivates cessation for the gifted. We were able to identify specific traits that can be connected to smoking triggers and inform cessation support. However, we did not expect to standardize and establish cut-off points for the OEQ-II specific to the Greek population nor to establish statistical significance for differences between groups in this study, due to the size of the sample. However, significant differences were observed in some areas, and there was convergence between the quantitative and qualitative results. Further research across a broader range of contexts, and with a non-gifted comparative sample, is now needed; this would be especially helpful in testing and refining the triggers questionnaire. The newly designed questionnaire was piloted in this study to collect nominal data, but a larger sample is needed to assess test-retest reliability, content validity, and construct validity. There are a range of methodologies for identifying gifted adults in the field but where counselling or support services are concerned, a person centered process of self-identification is often used^12,16,42-44^. Therefore, due to the focus on inner experience and building self-knowledge in this research, it was important to avoid IQ or other forms of intelligence testing in building relationships and trust with participants. This meant that we chose an invitational approach with self-identified gifted adults. While this approach was a strength in ensuring the person-centered approach to interviews was effective, it made comparative analysis with other studies challenging. This approach may also have introduced bias in terms of the nature of the sample, by excluding gifted individuals who could be identified using other methodologies and potentially different experiences of the relationship between overexcitabilities and smoking behaviors.

## CONCLUSIONS

This psychometric and person-centered interview approach to building self-knowledge about areas of hyperstimulation and smoking habits has the potential to improve outcomes for smoking cessation among some within the gifted population and those with multiple overexcitabilities. With further research, the OEQ-II and triggers questionnaires can be used in combination with other psychometric evaluations and with support aimed at the unique circumstances of each individual, so that an alternative pathway to smoking cessation is available.

## Data Availability

The data supporting this research are available from the authors on reasonable request.
