# Supplementary material for: Studying stimuli and smoking behaviors among self-identified gifted smokers and strategies for customizing cessation support
Source: Tob Induc Dis. 2022 Mar 8;20:20. doi: 10.18332/tid/143323 (PMC8899801; doi:10.18332/tid/143323)
Supplement: Supplementary file 1 [file TID-20-28-s1.pdf]

SUPPLEMENTARY FILE.

Supplementary Table 1. Descriptive characteristics of the study population and questionnaire completion (n=123

	Greek participants	Non-Greek participants	Total
All participants who completed at least the OEQ-II questionnaire	100	23	123
Participants who completed all tests	47	15	62
Participants who completed the first 3 tests	10	0	10
Participants who completed only one (the OEQ-II)	43	8	51
Participants with 3-5 overexcitabilities who completed all tests	39	9	48
Participants with 0-2 overexcitabilities who completed all tests	8	6	14

© 2022 Deligeorges D. et al.
